# Association of leptin and leptin receptor polymorphisms with coronary artery disease in a North Chinese Han population

**DOI:** 10.1590/0037-8682-0388-2019

**Published:** 2020-02-07

**Authors:** Haidong Wang, Chao Wang, Wenxiu Han, Chunmei Geng, Dan Chen, Bin Wu, Jun Zhang, Changshui Wang, Pei Jiang

**Affiliations:** 1 Phase I Clinical Trial Centre, The Affiliated Lianyungang Hospital of Xuzhou Medical University/The First People’s Hospital of Lianyungang, China.; 2 Department of Pharmacy, Hainan General Hospital, Haikou 570311, Hainan Province, China.; 3 Affiliated Jining First People's Hospital of Jining Medical University, China.; 4 Department of Gynecology, Taian City Central Hospital, Taian, Shandong, P.R. China.; 5 Department of Pharmacy, The First Affiliated Hospital of Zhengzhou University, Zhengzhou, China.

**Keywords:** Coronary artery disease, Leptin, Leptin receptor, Polymorphisms

## Abstract

**INTRODUCTION:**

Leptin (LEP) is a peptide hormone that acts via leptin receptor (LEPR) binding. Genetic evidence from different human populations has implicated LEP/LEPR in the pathogenesis of coronary artery disease (CAD), and suggests that certain LEP/LEPR gene polymorphisms may increase the risk of CAD. The aim of this study was to assess two single nucleotide polymorphisms (SNPs) in LEP genes (rs2167270 and rs7799039) and two in LEPR genes (rs6588147, rs1137100) for association with CAD.

**METHODS:**

We enrolled 271 North Chinese Han CAD patients, and 113 healthy age- and sex-matched controls. Genomic DNA was extracted from whole blood, and the four SNPs were assessed using a MassArray system.

**RESULTS:**

The G allele frequency at rs2167270 was significantly higher among CAD cases than among controls. The AG genotype at rs7799039 was associated with a significantly decreased risk of CAD unlike the AA genotype used as the reference. The A allele was significantly associated with the CAD patient group. Interestingly, statistically significant differences in genotype and allele frequency at *LEP* rs2167270 and rs7799039 existed among females but not among males.

**CONCLUSIONS:**

The current study detected a significant association between genetic variations at *LEP* rs7799039 and rs2167270 and the risk of CAD in a north Chinese population, and revealed that LEP rs2167270 and rs7799039 gene polymorphisms might act as predisposing factors for CAD.

## INTRODUCTION

Coronary artery disease (CAD) is the most common cardiovascular disease, and is characterized by reduced arterial blood flow to heart muscle. Worldwide, CAD is the most common human disease, with the highest morbidity and mortality, affecting 110 million people, and resulting in 8.9 million deaths in 2015 (up to 15.6% of all deaths)^1^. Risk factors for CAD include diabetes, high blood pressure, smoking, poor diet, and lack of exercise^2^. 

Leptin (LEP) is a peptide hormone discovered in 1994, and named for the Greek word leptos, which means “thin”^3^. The LEP gene is located on chromosome 7. It encodes a 167 amino acid peptide with a molecular weight of 16 kD. LEP is predominantly secreted in white adipose tissue, and functions by binding to the leptin receptor (LEPR)^4^. In recent years, LEP has been found to regulate energy balance in coordination with regulation of glucose and lipid metabolism. Thus, it plays a vital role in development of CAD^5^ ^,^ ^6^. 

An ample body of evidence from studies among different human populations has revealed that serum or plasma LEP levels correlate with CAD, suggesting that LEP/LEPR might be involved in CAD pathogenesis, and LEP/LEPR gene polymorphisms may influence the risk of CAD^7^ ^,^ ^8^. Studies to date of relationships between LEP/LEPR gene polymorphisms and CAD have produced varying results. Roszkowska-Gancarz^9^ reported that polymorphisms at LEP rs7799039 and LEPR rs1137100 were associated with risk of myocardial infarction, a type of CAD, in a Polish population. Similarly, A alleles at LEP rs7799039 and LEPR rs1137100 have been proposed to be risk factors for cardiovascular disease in Chinese populations^10^ ^,^ ^11^. However, a meta analysis^12^ did not find any association between the rs1137100 polymorphism and CAD. Results from Spain and Southern China did not show a significant association between LEP rs2167270 SNP and CAD^13^ ^,^ ^14^. In addition, research on LEPR rs6588147 has mainly focused on the correlation between its polymorphism and the risk of cancer. Any association between rs6588147 SNPs and CAD has rarely been reported.

Given the inconsistency of results from studies among different races, this study aims to evaluate four SNPs (rs2167270 (G>A), rs7799039 (A>G), rs6588147 (G>A), and rs1137100 (G>A)) in the LEP and LEPR genes in patients with CAD in a North Chinese population to provide a frame of reference for related evaluations.

## METHODS

### Subjects

All participants in this study were recruited from the First Peoples’ Hospital of Jining between February 2016 and May 2018. The study enrolled 271 north Chinese Han patients with CAD. All patients with CAD were diagnosed by experienced cardiologists based on the following criteria: angiographic evidence of luminal diameter constriction > 50% in at least one main coronary artery, previous history of coronary artery bypass graft surgery, or a history of percutaneous coronary intervention. Among the patients with CAD, those who had a diastolic blood pressure ≥ 90 mmHg and/or a systolic blood pressure ≥ 140 mmHg, or were taking antihypertensive drugs, were defined as having hypertension. Patients with renal failure, congenital heart disease, tumors, immune system disorders, malignancies, congenital heart disease, or infectious heart disease were excluded. The 113 healthy age- and sex-matched controls were selected from among physical examination program participants based on electrocardiogram results at the time of clinical examination. Chest radiography, questionnaires, and medical history evaluation were employed to identify control subjects free from CAD and hypertension. Data from physicians’ reports and medical records were used to identify qualified controls. 

This study was designed in accordance with the Declaration of Helsinki, and approved by the ethics committee of the First Peoples’ Hospital of Jining (approval number: JY2016011). All subjects provided written informed consent.

DNA Isolation and Genotyping: About 1 ml of peripheral blood was collected and extracted from each subject using a TIANamp Blood DNA Kit (TIANGEN, China) according to the manufacturer’s instructions. Concentration and purity of DNA samples were assessed with a NanoDrop-1000 (NanoDrop, USA) to ensure that enough sample would be available for subsequent experiments. All DNA samples were genotyped by polymerase chain reaction (PCR)-ligase detection reaction. Sequences from each participant containing the four target single-nucleotide polymorphisms were amplified using the primers listed in Table 1. Samples were dephosphorylated with shrimp alkaline phosphatase, extended, and purified using iPLEX extension reagents (Agena Bioscience, USA) and a Nanodispenser RS1000, respectively. Matrix-assisted laser desorption/ionization time-of-flight mass spectrometry was conducted to identify primer extension products. The software Spectro-TYPER was used to automatedly analyze the genotyping data. More than 10% of the samples were randomly selected and retested to validate the MassARRAY results.

**TABLE 1:** PCR Primers targeting LEP/LEPR Gene SNPs in this study

SNP	Ancestral allele	Primer sequence	Product size
rs2167270	G	5’-CCAGGAAAAGCCTGTCACAT-3’	250
		3’-CTGGCAGAGCGACTAAAAGC-5’	
rs7799039	A	5’-CCAGGAAAAGCCTGTCACAT-3’	250
		3’-CTGGCAGAGCGACTAAAAGC-5’	
rs6588147	G	5’-TTCCACTGGCAAAACACATT-3’	186
		3’-CAGCTGGGAAACTTTTCATCAT-5’	
rs1137100	G	5’-ATGTTTTTGGCAACCCAGAG-3’	211
		3’-GTAGAGACGGGGTTTCACCA-5’	

### Statistical Analysis

All genotyping results from patients and controls were tested for Hardy-Weinberg equilibrium (HWE) by chi-square (χ^2^) analysis.

Differences in genotype distributions and allele frequencies between case and control groups were evaluated for statistical significance by chi-square (χ^2^) analyses. Logistic regression analysis adjusted by age was used to evaluate associations between each polymorphism and risk of CAD. Associations between genotypes and CAD were evaluated via the odds ratio (OR), with a 95% confidence interval (95% CI). The significance level was set at 0.05. All statistical analyses were performed with SPSS 17.0 for Windows (SPSS Inc., Chicago, IL, USA).

## RESULTS

No statistically significant differences in age or sex were found between the CAD and control groups. Mean ± SD ages were 54.31 (9.29) years for the CAD group, and 52.49 (7.68) years for the control group (P = 0.068). The percentages of men and women were 47.2% and 52.8%, respectively, in the CAD group, and 46.9% and 53.1% among the controls (P = 0.953). Control group systolic (SBP) and diastolic blood pressures (DBP) were 126.51 (12.26) mmHg and 74.22 (7.67) mmHg, respectively, significantly lower than those of the CAD group (147.31 (9.26) mmHg SPB, and 89.74 (10.91) mmHg DBP).

The genotype distribution and allele frequencies of the CAD and control groups at the four genetic polymorphism sites were compared, and the results are shown in Table 2. The four observed genotype frequencies were in accordance with HWE. Distributions of the four LEP genotypes and alleles among male and female CAD patients and controls are shown in Table 3 and Table 4, respectively. 

**TABLE 2:** Comparison of Genotypic and Allelic Distribution of LEP rs2167270 and rs7799039, and LEPR rs6588147 and rs1137100 SNPs between all Patients (n=271) and Controls (n =113).

SNP	Genotype/ allele	Case,(%)	Control,(%)	***P* value** ^a^ **(χ** ^2^ **)**	OR (95% CI)	***P* value** ^b^
rs2167270	GG	195 (72.0)	71 (62.8)	0.142 (3.903)	1.00	Referent
	GA	69 (25.5)	36 (31.9)		0.698 (0.429-1.135)	0. 147
	AA	7 (2.5)	6 (5.3)		0.425 (0.138-1.307)	0.135
	GA+AA	76 (28.0)	42 (37.2)	0.077 (3.119)	0.659 (0.414-1.048)	0.078
	G	459 (84.7)	178 (78.8)	0.047 (3.958)*	1.00	Referent
	A	83 (15.3)	48 (21.2)		0.671 (0.452-0.996)	0.048*
rs7799039	AA	170 (62.7)	52 (46.0)	0.010 (9.143)*	1.00	Referent
	AG	94 (34.7)	57 (50.5)		0.504 (0.321-0.793)	0.003*
	GG	7 (2.6)	4 (3.5)		0.535 (0.151-1.901)	0.334
	AG+GG	103 (37.3)	61 (54.0)	0.003 (9.133)*	0.506 (0.325-0.790)	0.003*
	A	434 (80.1)	161 (71.2)	0.008 (7.134)*	1.00	Referent
	G	108 (19.9)	65 (28.8)		0.616 (0.431-0.881)	0.008*
rs6588147	GG	197 (72.7)	82 (72.6)	0.958 (0.086)	1.00	Referent
	GA	68 (25.1)	29 (25.7)		0.976 (0.589-1.618)	0.925
	AA	6 (2.2)	2 (1.8)		1.249 (0.247-6.316)	0.788
	GA+AA	74 (27.3)	31 (55.7)	0.980 (0.001)	0.994 (0.607-1.625)	0.980
	G	462 (85.2)	193 (85.4)	0.995 (0.003)	1.00	Referent
	A	80 (14.8)	33 (14.6)		1.013 (0.653-1.571)	0.995
rs1137100	GG	188 (69.4)	75 (66.4)	0.837 (0.357)	1.00	Referent
	GA	75 (27.7)	34 (30.1)		0.880 (0.541-1.430)	0.606
	GG	8 (2.9)	4 (3.5)		0.798 (0.233-2.729)	0.719
	GA+GG	83 (30.6)	38 (33.6)	0.564 (0.333)	0.871 (0.546-1.391)	0.564
	G	451 (83.2)	184 (81.4)	0.549 (0.359)	1.00	Referent
	A	91 (16.8)	42 (15.6)		0.884 (0.590-1.324)	0.549

**CI:** confidence interval; **OR:** odds ratio. ^a^ *P* value for genotype and allele frequencies in cases and controls using 2-sided χ^2^ tests.^b^ *P* values adjusted by age and gender using logistic regression. *P<0.05.

**TABLE 3:** Comparison of Genotypic and Allelic Distributions of LEP rs2167270 and rs7799039, and LEPR rs6588147 and rs1137100 SNPs Between Male Patients (n=128) and Controls (n =53).

SNP	Genotype/ allele	Case,(%)	Control,(%)	***P* value** ^a^ **(χ** ^2^ **)**	OR (95% CI)	***P* value** ^b^
rs2167270	GG	95 (74.3)	39 (73.6)	0.927 (0.056)	1.00	Referent
	GA	30 (23.4)	13 (24.5)		0.947 (0.448-2.006)	0.888
	GG	3 (2.3)	1 (1.9)		1.232 (0.124-12.206)	0.859
	GA+AA	33 (25.7)	14 (26.4)	0.929 (0.008)	0.968 (0.467-2.004)	0.929
	G	220 (85.9)	91 (85.8)	0.708 (0.140)	1.00	Referent
	A	36 (14.1)	15 (14.2)		1.129 (0.598-2.133)	0.708
rs7799039	AA	76 (59.4)	27 (50.9)	0.187 (3.349)	1.00	Referent
	AG	48 (37.5)	26 (49.1)		0.656 (0.343-1.255)	0.202
	GG	4 (3.1)	0 (0.0)		-------	------
	AG+GG	52 (40.6)	26 (49.1)	0.297 (1.087)	0.711 (0.373-1.353)	0.298
	A	200 (78.1)	80 (75.5)	0.583 (0.301)	1.00	Referent
	G	56 (21.9)	26 (24.5)		0.862 (0.506-1.467)	0.583
rs6588147	GG	94 (73.4)	39 (73.6)	0.982 (0.037)	1.00	Referent
	GA	31 (24.3)	13 (24.5)		0.989 (0.469-2.089)	0.978
	AA	3 (2.3)	1 (1.9)		1.245 (0.126-12.337)	0.852
	GA+AA	34 (26.6)	14 (26.4)	0.984 (0.000)	1.008 (0.488-2.082)	0.984
	G	219 (85.5)	91 (85.8)	0.941 (0.004)	1.00	Referent
	A	37 (14.5)	15 (14.2)		1.025 (0.536-1.959)	0.941
rs1137100	GG	89 (69.5)	38 (71.7)	0.273 (2.599)	1.00	Referent
	GA	33 (25.8)	15 (28.3)		0.919 (0.458-1.928)	0.864
	GG	6 (4.7)	0 (0.0)		------	----
	GA+GG	39 (30.5)	15 (28.3)	0.772 (0.084)	1.110 (0.548-2.250)	0.772
	G	211 (82.4)	91 (85.8)	0.425 (0.637)	1.00	Referent
	A	45 (17.6)	15 (14.2)		1.294 (0.686-2.439)	0.426

**CI:** confidence interval; **OR:** odds ratio. ^a^ *P* value for genotype and allele frequencies in cases and controls using 2-sided χ^2^ test.^b^ *P* values adjusted by age using logistic regression.

**TABLE 4:** Comparison of Genotypic and Allelic Distribution of LEP rs2167270 and rs7799039, and LEPR rs6588147 and rs1137100 SNPs Between Female Patients (n=143) and Controls (n =60).

SNP	Genotype/ allele	Case (%)	Control (%)	***P* value** ^a^ **(χ** ^2^ **)**	OR (95% CI)	***P* value** ^b^
rs2167270	GG	100 (70.0)	32 (53.3)	0.041 (6.405)*	1.00	Referent
	GA	39 (27.2)	23 (38.4)		0.543 (0.283-1.041)	0.066
	AA	4 (2.8)	5 (8.3)		0.256 (0.065-1.011)	0.052
	GA+AA	43 (30.3)	28 (46.7)	0.024 (5.119)*	0.491 (0.264-0.914)	0.025*
	G	239 (83.6)	87 (72.5)	0.011 (6.543)*	1.00	Referent
	A	47 (16.4)	33 (27.5)		0.518 (0.312-0.862)	0.011*
rs7799039	AA	94 (65.7)	25 (41.7)	0.004 (0.972)*	1.00	Referent
	AG	46 (32.2)	31 (51.7)		0.395 (0.209-0.744)	0.004*
	GG	3 (2.1)	4 (6.6)		0.199 (0.042-0.950)	0.043*
	AG+GG	49 (34.3)	35 (58.3)	0.001 (10.093)*	0.372 (0.201-0.691)	0.002*
	A	234 (81.8)	81 (67.5)	0.002 (9.965)*	1.00	Referent
	G	52 (18.2)	39 (32.5)		0.462 (0.284-0.750)	0.002*
rs6588147	GG	103 (72.0)	43 (71.7)	0.975 (0.051)	1.00	Referent
	GA	37 (25.9)	16 (26.6)		1.375 (0.750-2.523)	0.920
	AA	3 (2.1)	1 (1.7)		0.904 (0.373-2.189)	0.847
	GA+AA	40 (28.0)	17 (28.3)	0.958 (0.004)	0.982 (0.503-1.919)	0.958
	G	243 (85.0)	102 (85.0)	0.993 (0.000)	1.00	Referent
	A	43 (15.0)	18 (15.0)		1.003 (0.552-1.821)	0.993
rs1137100	GG	99 (69.2)	37 (61.6)	0.111 (4.404)	1.00	Referent
	GA	42 (29.4)	19 (31.7)		0.826 (0.427-1.599)	0.571
	GG	2 (1.4)	4 (6.7)		0.187 (0.033-1.063)	0.059
	GA+GG	44 (30.8)	23 (38.3)	0.296 (1.094)	0.715 (0.381-1.343)	0.297
	G	240 (83.9)	93 (77.5)	0.125 (2.360)	1.00	Referent
	A	46 (16.1)	27 (22.5)		0.660 (0.388-1.124)	0.126

**CI:** confidence interval; **OR:** odds ratio. ^a^ *P* value for genotype and allele frequencies in cases and controls using 2-sided χ^2^ test. ^b^ *P* values adjusted by age using logistic regression. *P< 0.05.

No statistically significant difference was observed between CAD patients and controls in genotypes and allele distributions at LEPR rs6588147 (G>A) and rs1137100 (G>A) polymorphism sites.

The G allele frequency at LEP rs2167270 was significantly higher among CAD cases than among controls (P = 0.047). For LEP rs7799039, when the AA genotype was used as a reference, the AG genotype was associated with a significantly decreased risk of CAD (OR = 0.504, 95% CI = 0.321-0.793, p = 0.003), and the GG genotype was not significantly associated with any CAD risk (OR = 0.535, 95% CI = 0.151-1.901, p = 0.334). Moreover, the A allele showed a significant association with the CAD group (OR = 0.616, 95% CI = 0.431-0.881, p = 0.008). 

Interestingly, a statistically significant difference in the rs7799039 (A>G) genotype and allele frequency only existed among females (OR =0.395, 95% CI = 0.209-0.744, p = 0.004 for AG vs. AA; OR =0.199, 95% CI = 0.042-0.950, p = 0.043 for GG vs. AA; OR =0.372, 95% CI =0.201-0.691, p = 0.002 for AG+GG vs. AA; OR =0.462, 95% CI =0.284-0.750, p = 0.002 for G vs. A). No statistically significant difference was found among males. A similar difference was also observed in the rs2167270 (G>A) genotype and allele frequency among females (OR =0.491, 95% CI =0.264-0.914, p = 0.025 for GA+AA vs. GG; OR =0.518, 95% CI =0.312-0.862, p = 0.011 for A vs. G). 

CAD patients were subgrouped ± hypertension (CAD+H^+^ for patients with CAD and hypertension, and CAD+H^-^ for patients with CAD but no hypertension), and the genotypes and allele frequencies of the two subgroups were compared with those of the controls. The results are presented in Table 5. No significant allelic or genotypic associations were found between the rs6588147 and rs1137100 SNPs and CAD in either subgroup (P=0.085-0.976). Both subgroups showed significant differences from the control group (p=0.005 and 0.031 for CAD+H+ and CAD+H-, respectively) in AG/AA genotype ratio at rs7799039. The frequency of genotypes with G (AG + GG) was significantly different among CAD+H^+^ and CAD+H^-^ patients compared to that in the controls: p = 0.005 and 0.024 for CAD+H^+^ and CAD+H^-^, respectively. Moreover, the A allele showed significantly stronger associations with CAD+H^+^ and CAD+H^-^ groups than with the control group (p=0.014 and 0.042 for CAD+H^+^ and CAD+H^-^, respectively). The G/A genotype of LEP rs2167270 showed a significant association with CAD+H^+^ compared to that observed in the control group (p=0.045), but showed no significant association with CAD+H^-^.

**TABLE 5:** Genotypic/Allele Distribution of LEP rs2167270 and rs7799039, and LEPR rs6588147 and rs1137100 SNPs Between Coronary Artery Disease patients with (n=210) and Coronary Artery Disease Patients without Hypertension (n=61), and Healthy Controls (n =113).

SNP	Genotype/Allele	CAD+H^+^ (%)	CAD+H^-^ (%)	Control,(%)	***P* value**			
					CAD+H^+^ *vs* Control		CAD+H^-^ *vs.* Control	
rs2167270	GG	152 (73.4)	43 (70.5)	71 (62.8)	0.136	Referent	0.568	Referent
	GA	53 (25.2)	16 (26.2)	36 (31.9)		0.148		0.386
	GG	5 (2.4)	2 (3.3)	6 (5.3)		0.118		0.471
	GA+AA	58 (27.6)	18 (29.5)	42 (37.2)		0.077		0.310
	G	357 (85.0)	102 (83.6)	178 (78.8)		Referent		Referent
	A	63 (15.0)	20 (16.4)	48 (21.2)		0.045*		0.277
rs7799039	AA	131 (62.4)	39 (63.9)	52 (46.0)	0.018*	Referent	0.074	Referent
	AG	73 (34.8)	21 (34.5)	57 (50.5)		0.005*		0.031*
	GG	6 (2.8)	1 (1.6)	4 (3.5)		0.432		0.313
	AG+GG	79 (37.6)	22 (36.1)	61 (54.0)		0.005*		0.024*
	A	335 (79.8)	99 (81.1)	161 (71.2)		Referent		Referent
	G	85 (20.2)	23 (18.9)	65 (28.8)		0.014*		0.042*
rs6588147	GG	148 (70.5)	49 (80.3)	82 (72.6)	0.890	Referent	0.516	Referent
	GA	57 (27.1)	11 (18.1)	29 (25.7)		0.749		0.251
	AA	5 (2.4)	1 (1.6)	2 (1.8)		0.700		0.885
	GA+AA	62 (29.5)	12 (19.7)	31 (55.7)		0.692		0.257
	G	353 (84.0)	109 (89.3)	193 (85.4)		Referent		Referent
	A	67 (16.0)	13 (10.7)	33 (14.6)		0.651		0.300
rs1137100	AA	141 (67.1)	47 (77.0)	75 (66.4)	0.976	Referent	0.171	Referent
	GA	61 (29.1)	14 (23.0)	34 (30.1)		0.856		0.252
	GG	8 (3.8)	0 (0.0)	4 (3.5)		0.922		0.117
	GA+GG	69 (32.9)	14 (23.0)	38 (33.6)		0.888		0.142
	G	343 (81.7)	108 (88.5)	184 (81.4)		Referent		Referent
	A	77 (18.3)	14 (11.5)	42 (18.6)		0.938		0.085

*P* values for genotype frequencies in cases and controls using 2-sided χ^2^ test. *P< 0.05.

CAD+H^+^ for Coronary Artery Disease Patients with Hypertension.

CAD+H^-^ for Coronary Artery Disease Patients without Hypertension.

## DISCUSSION

CAD is a prominent global cause of morbidity and mortality, leading to 17.3 million deaths per year worldwide, and its prevalence continues to increase^15^. In 2017, a summary reported 290 million people with cardiovascular disease in China, of whom 11 million suffered from CAD, causing immense suffering and placing a heavy burden on healthcare services^16^. 

The exact pathogenesis of CAD remains unclear. Relative risk factors include hypertension, smoking, dyslipidemia, diabetes, being overweight, and obesity. The best known functions of LEP are appetite reduction, enhancement of energy expenditure, and regulation of lipid metabolism, all of which contribute to regulating obesity, an important CAD risk factor^8^. Certain levels of plasma or serum LEP can exert other physiological effects, such as enhancing endothelial cell oxidative stress, stimulating growth of vascular smooth muscle cells, inducing arterial vascular wall injury, stimulating formation of reactive oxygen species, and stimulating the renin-angiotensin-aldosterone system^17^ ^,^ ^18^ All of these effects may influence development of CAD.

We investigated possible associations of four LEP/LEPR polymorphisms with CAD susceptibility in a north Chinese population. Our results demonstrate that LEPR rs6588147 and LEPR rs1137100 polymorphisms are not associated with CAD within the studied population. However, we found that LEP rs2167270 and LEP rs7799039 gene polymorphisms do influence CAD risk.

To date, research on rs6588147 is limited, and has mainly focused on correlating the LEPR rs6588147 polymorphism with risk of cancer. We failed to detect a significant association between rs6588147 SNPs and CAD risk in this study.

Previous studies have reported an association between rs1137100 polymorphisms and CAD and related diseases. Aijälä M^19^ reported a significant protective impact of the rs1137100 genotype against CAD, and proposed that the rs1137100 genotype of LEPR protects from adverse cardiovascular events. An^10^ et al. reported that the A allele in the rs1137100 polymorphism may be an independent risk factor for coronary atherosclerosis in Chinese patients with non-alcoholic fatty liver disease. Roszkowska-Gancarz M.’s^9^ study showed that the LEPR rs1137100 polymorphism significantly differs between centenarians and myocardial infarction groups. However, this study did not find any association between the rs1137100 polymorphism and CAD, consistent with the results of a previous meta analysis^12^, which included 1989 cases and 2601 controls, and found no potential associations of the LEPR rs1137100 variant with CAD. 

Our study showed an association between the LEP rs2167270 A allele and CAD, which differs from the results of previous studies. García-Bermúdez M et al.^13^ showed that the LEP rs2167270 polymorphism is not a genetic risk factor for disease susceptibility or clinically evident CV disease. Tan^14^ conducted a large population-based study in southern China, including 1044 CAD patients and 1349 healthy individuals, but did not find any significant association between LEP rs2167270 SNP and CAD in the southern Chinese Han population.

Extensive research has been conducted on associations between LEP rs7799039 polymorphisms and CAD, but the results have been inconsistent. Zohreh Nowzari^20^ searched for associations between LEP and LEPR gene polymorphisms and CAD susceptibility and hypertension among Iranians. They reported that the LEP rs7799039 gene polymorphism does not serve as a predisposing factor for CAD in the studied Iranian population. Roszkowska-Gancarz^9^ reported that the GG genotype at the rs7799039 LEP polymorphism is significantly more common among centenarians than among myocardial infarction patients. Zhao^11^ reported that the LEP polymorphism rs7799039 significantly correlates with CAD, and that the A allele may contribute to individual CAD susceptibility among a population in Tianjin, China. The subjects of our research were drawn from Shandong Province, which is near Tianjin. We obtained similar results, indicating that the LEP rs7799039 variant is significantly associated with CAD, also consistent with the results of the meta-analysis by Xiao^16^.

Based on the above-mentioned studies, some results from different reports have been inconsistent or contradictory to our results regarding associations of LEP and LEPR polymorphisms with CAD. The main reason for this difference may be that all these results come from different races. Even the Chinese samples were obtained from different areas of China. We compared the minor allele frequency (MAF) in our study with data from the NCBI database (data no shown), and found that our results are consistent with data from a Vietnamese population, but very different from data obtained from a western population. We further analyzed data from different literature sources, and found similar phenomena. Thus, the effects of LEP and LEPR polymorphisms on CAD may vary significantly among different races.

Hypertension has been found to be a strong independent risk factor for CAD, so we further stratified our analyses by hypertension. These results demonstrated that the LEP rs7799039 genotype and allele are significantly associated with CAD, regardless of whether the patient has hypertension or not. By contrast, the LEP rs2167270 allele was only significantly associated with CAD plus hypertension. LEP rs7799039 may independently affect the incidence of CAD, whereas LEP rs2167270 may affect the incidence of CAD in concert with hypertension.

We discovered the intriguing phenomenon that significant associations of LEP rs2167270 and rs7799039 with CAD were only found among females. LEP concentrations are higher per kilogram of body weight in women than in men. This difference is eliminated after adjusting for circulating concentrations of sex hormones^21^. LEP production is upregulated by estrogens, and downregulated by androgens^22^. LEP expression in adipocytes can also be upregulated by estrogen^23^. Another study has shown that LEP rs7799039 is significantly associated with increased risk for gestational weight gain. Thus it may affect other weight related diseases among females^24^. 

This study has some limitations. First, this study analyzed a limited size population and was confined to northern China. Our power analyses based on sample size and minor allele frequencies of the studied SNPs (Data not shown) indicated that our study could not achieve 80% power, placing a limitation on this study. Larger sample sizes including diverse groups are needed for more reliable outcomes. Second, many factors affect the pathogenesis of CAD, including smoking history, diabetes mellitus, and blood lipid level. We failed to adjust for these factors due to limited data. Third, this study only included four genotypes of LEP and LEPR, which does not fully assess potential associations of LEP and LEPR polymorphisms with CAD.

## CONCLUSION

In conclusion, the current study revealed significant associations between LEP rs7799039 and rs2167270 genetic variations and the risk of CAD in a north Chinese population. These findings suggest that replication studies are necessary in ethnically disparate populations, assessing variants spanning the gene.
